# Influence of Vegetarian Dietary Intervention on Urinary Paraben Concentrations: A Pilot Study with ‘Temple Stay’ Participants

**DOI:** 10.3390/toxics8010003

**Published:** 2020-01-17

**Authors:** Areum Jo, Sunmi Kim, Kyunghee Ji, Younglim Kho, Kyungho Choi

**Affiliations:** 1 Department of Environmental Health Sciences, School of Public Health, Seoul National University, Seoul 08826, Korea; jojo721@korea.kr (A.J.); sonmilove@gmail.com (S.K.); 2 Accident Prevention and Assessment Division II, National Institute of Chemical Safety, Daejeon 34111, Korea; 3 Institute of Health and Environment, Seoul National University, Seoul 08826, Korea; 4 Department of Occupational and Environmental Health, Yongin University, Yongin 17092, Korea; kyungheeji@yongin.ac.kr; 5 School of Human and Environmental Sciences, Eulji University, Seongnam 13135, Korea; ylkho@eulji.ac.kr

**Keywords:** parabens, dietary intervention, exposure source, temple stay, Korea

## Abstract

Personal care products and cosmetics have been identified as major sources of paraben exposure among humans. However, the contribution of dietary factors has not been well understood. We recruited temple stay participants (*n* = 25) who followed a strict Buddhist vegetarian diet during a five-day period, and assessed the influence of this lifestyle change, employing their urine samples collected before and after the temple stay. Before the temple stay, methylparaben (MeP) was detected at the highest levels, followed by ethylparaben (EtP), propylparaben (PrP), butylparaben (BuP), and benzophenones (BPs) in the urine samples. Following the temple stay, the urinary EtP concentrations remarkably increased from 14.0 to 105 μg/L, and were around two orders of magnitude higher than those reported from other countries. Dietary factors associated with the temple diet may partly explain the increase, because EtP is allowed in Korea for seasoning and condiments, which are frequently added in vegetarian diets. Following the temple stay, however, MeP, PrP, and BPs did not show significant decreasing trends. In contrast, BuP levels decreased significantly, especially in male urine samples, that is, from 3.60 to 1.03 μ/L, suggesting a reduced use of certain personal care products during the temple stay. Our observations outline the potential importance of dietary factors on EtP exposure, and might help explain its high exposure levels among Korean population.

## 1. Introduction

Parabens (esters of p-hydroxybenzoic acid) have been widely used as antimicrobials in various applications including cosmetics, personal care products (PCPs), and food items [1,2,3,4]. Among them, methylparaben (MeP), ethylparaben (EtP), propylparaben (PrP), and butylparaben (BuP) have been most frequently used [5]. In experimental studies, several parabens have been demonstrated to influence the male reproductive system including spermatogenesis, sperm mobility, and reproductive organ weight [6,7,8]. Moreover, limited human observational studies suggest that some parabens are linked to adverse effects on oxidative stress, fecundity, and gestation age [9,10,11].

Parabens are non-persistent compounds that have short half-lives of about 4–8 h in the human body [12]. While urinary paraben levels fluctuate over time, humans are consistently exposed to parabens through numerous sources in daily lives [13,14]. Several nation-wide biomonitoring programs have shown the widespread occurrences of these chemicals in humans. Among the general population of the USA participating in the National Health and Nutrition Examination Survey 2011–2012 (*n* = 2489; [15]), both MeP and PrP have been detected in >94% of the population. Of 660 urine samples collected in Germany between 1995 and 2012, MeP, EtP, and PrP were detected in 79–99% [16,17]. In most cases, MeP has been detected at the highest concentrations, followed by PrP, EtP, and BuP [18,19].

Personal care products are among the most widely recognized sources of paraben exposure among the general population. For example, MeP and PrP have been frequently detected (>60%) in hand cream (on average, 2826 μg/g), body lotion (1564 μg/g), and sunscreen (1360 μg/g) in China [3] and the U.S.A. [2]. Parabens are also permitted as preservatives in processed foods and baked goods [20]. However, contribution of diet on paraben exposure is not well characterized, mostly because of a lack of measurement data. Currently available information indicates that levels of parabens in food are relatively lower compared with those measured in PCPs [21]. In one study, contribution of food intake is estimated at 2.6%~5.5% and 0.42% of the total paraben exposure among general populations of China and the U.S.A., respectively [22].

In the Korean population, parabens are also frequently detected in urines. Unlike other countries, however, EtP is detected at higher levels in the urine of Korean people [15,23,24,25], which are generally >10 times higher compared with those observed in populations of other countries such as China, India, Belgium, Denmark, and the U.S.A., that is, on average, around 30 μg/L among Korean versus <2 μg/L in other countries [23,26,27,28]. Though potential contribution from dietary sources has been suggested [15], information on exposure sources and pathways of EtP among the Korean population is not available.

This pilot study was conducted to identify major exposure sources of parabens with a focus on EtP, employing an intervention study design on a small adult population. For this purpose, a group of adult participants who took part in a five-day temple stay program was employed. During the temple stay program, the participants followed a strict vegetarian Buddhist diet, and daily routines of the monks, which included meditation and pray. Details about this population and the temple stay program have been reported elsewhere [29]. Along with four parabens, two benzophenones, that is, benzophenone-3 (BP-3) and its major metabolite, benzophenone-1 (BP-1), were measured in the urine samples, because benzophenones are expected to be more exclusively used in personal care products than in dietary items [30,31]. The results of this study are expected to improve current understanding of the dietary contribution to the paraben exposure among Koreans, and to help develop an appropriate exposure mitigation program.

## 2. Materials and Methods

### 2.1. Study Population and Sample Collection

A total of 30 participants who participated in a temple stay program in Geumsan Temple (Gimje, Korea) for five days in August 2007 were recruited. Because of limited availability of urine samples, 25 participants were chosen and their urine samples were analyzed in the present study (Table S1). The subjects were between 13 and 64 years of age with 16 males and 9 females (Table S1). None of the participants declared to be vegetarian or vegan before the temple stay (Table S2). During the temple stay program, all the participants maintained a Buddhist vegetarian diet, kept away from instant or fast foods, and followed daily routines of monks. The Buddhist vegetarian diet in the present study did not include any meat, eggs, dairy, and fish products.

Urine samples were collected two times from all participating adults; first, within 1 h of the program participation (morning of day 1), and second, within two hours before the conclusion of the program (early afternoon of day 5). Urine samples were collected in 50 mL polypropylene tubes. Following sampling, urine was immediately transferred to the laboratory under cold condition, and stored at −20 °C until analysis. The present study was approved by the Institutional Review Board of School of Public Health, Seoul National University (Approval No. 2007-04-30-37, date of approval: 14 May 2007). Informed written consents was provided by all the participants.

### 2.2. Laboratory Analyses

The urine samples were treated and analyzed following the methods of Ye et al. [32] with some modifications. Target chemicals; internal standards, that is, MP-d4, EP-d4, PP-d4, and BP-d4; and β-glucuronidase (Helix pomatia, H1) were purchased from CDN Isotopes (Pointe-Claire, Quebec, Canada) and Sigma-Aldrich (St. Louis, MO, USA.), respectively. Briefly, 1 mL of the urine sample was mixed with 50 μL of 1 μg/mL internal standard solution, 50 μL of β-glucuronidase/arylsulfatase solution, and 1 M ammonium acetate buffer (pH 5). After a 4 h incubation at 37 °C, 730 μL of 0.1 M acetic acid was added. Solid phase extraction was performed using Sep-pak C18 cartridge (1 cc/100 mg, Waters, Milford, MA, USA). For the conditioning of the cartridge, 2 mL methanol and 2 mL water were added. Then, after sample loading, the cartridge was subsequently washed with 2 mL water and 2 mL methanol (5%), after which the target compounds were eluted by 1 mL methanol. Analytical column of Synergi 4U Fusion-RP (80A, 2.0 × 75 mm, Phenomenex, Torrance, CA, USA) in gradient mode was employed for separation, and target compounds were detected by API 4000 triple quadrupole mass spectrometer (Applied Biosystems, Foster City, CA, USA.) with multiple reaction mode (MRM). For LC/MS-MS condition and operational parameters for detection, refer to Supplementary Tables S3 and S4. Creatinine was measured using enzymatic method in Samkwang Medical Laboratories (Seoul, Korea).

Both the accuracy and precision of the analysis for each compound were within the acceptable range, that is, <20%. The recovery of all analytes in the quality control spiked samples ranged between 84.5% and 113.6% and the coefficient of variation (CV) was <20% (Table S5). The limits of detection (LODs) were 0.7 μg/L for MeP, 0.2 μg/L for EtP, 0.3 μg/L for PrP, 0.5 μg/L for BuP, 1 μg/L for BP-1, and 3 μg/L for BP-3.

### 2.3. Estimation of Daily Intake of Parabens

Daily intake of each paraben was estimated from the following formula [16,17].

EDI = UC × UV_24 h_/(F_ue_ × bw) (1)

where EDI is an estimated daily intake (μg/kg body-weight/day); UC is a urinary concentration (μg/L); UV_24 h_ is the total volume of urine in 24 h (assumed at 2 L); F_ue_ is the urinary excretion fraction, that is, MeP = 17.4, EtP = 13.7, PrP = 10.2, and BuP = 5.6 [16]; and bw is body weight. The EDI derived for each compound was compared with acceptable daily intake established by a relevant authority or a scientific literature [33,34].

### 2.4. Statistical Analyses

Urinary concentrations of target compounds were shown in both unadjusted (in μg/L) and creatinine-adjusted values (in μg/g creatinine). Both data were used for statistical analyses. Urinary levels of parabens and BPs were not normally distributed, and thus were converted to natural logs. Non-detects were substituted with the respective LOD was divided by the square root of 2 [35]. Wilcoxon signed-rank test was used to compare between the concentrations of urines collected pre- and post-intervention. For the Wilcoxon signed-rank test, creatinine-adjusted chemical concentrations were used to control dilution of the urine. Because we measured the levels of parabens and BPs in both pre- and post-intervention urine collected from the same people, mixed-effect models were used, with participant included as multilevel random effects, and age and log-transformed creatinine concentrations as fixed effects, in order to evaluate the changes associated with the intervention. As a sensitivity analysis to support the results of the mixed-effect model, the Wilcoxon signed-rank test was conducted. In addition, a mixed-effect model was conducted by gender classification, and its results were compared with those obtained from the Mann–Whitney U Test. All statistical tests were performed using R software (Version 3.2.1, The R Foundation, Vienna, Austria).

## 3. Results

### 3.1. Urinary Concentrations of Parabens and Benzophenones

All target parabens (MeP, EtP, PrP, and BuP) were detected in most urine samples (*n* = 25, Table 1). Before the temple stay, MeP was detected at the highest concentrations (geometric mean; GM 84.87 μg/L), followed by EtP (GM 14.03 μg/L), PrP (GM 11 μg/L), and BuP (GM 4.71 μg/L) among the participating adults. Following the temple stay, MeP was detected again at the highest concentrations (GM 109.11 μg/L), followed by EtP (GM 105.31 μg/L), PrP (GM 21.06 μg/L), and BuP (GM 2.06 μg/L). Regardless of the temple stay, females showed significantly higher levels of urinary MeP, PrP, and BuP compared with males (Table S6, mixed-effect model by gender classification). Especially, GMs of urinary PrP and MeP levels among female were up to >13 and >7 times higher compared with those of males, respectively (Table 1).

After the temple stay, EtP levels (GM) increased significantly by >9 and >6 times in male and female participants, respectively. Among the male participants, following temple stay, urinary EtP levels became higher than MeP, that is, GM 121.78 μg/L and 58.61 μg/g creatinine for EtP versus 53.74 μg/L, or 25.86 μg/g creatinine for MeP. In addition, a significant decrease of BuP levels was observed in male participants, that is, 3.60 versus 1.03 μg/L, following the intervention (Table 1). Both the mixed effect model and the paired Wilcoxon signed-rank test exhibited the same trend of the change following the temple stay (Tables S6 and S7).

BP-3 and BP-1 were detected in all samples except one, regardless of the intervention (Table 1). The GMs of the urinary BP concentrations were generally higher by three to four times among the females, but statistical significance between genders was not detected. For both BP-3 and BP-1, the intervention did not lead to any significant changes in the urinary concentration (Tables S6 and S7). However, following the temple stay, urinary levels (GM) of BP-3 decreased by >2 times in females, but such decrease was not observed among the male participants (Table 1).

### 3.2. Estimated Daily Intake of Parabens

EDIs (median) calculated for MeP, EtP, and BuP were 17.46, 37.17, and 0.52 μg/kg-d for male, and 224.58, 39.54, and 9.74 μg/kg-d for female participants, respectively (Table S11). For women, the EDIs calculated for MeP and EtP account for 2.24% and 0.40% of the group acceptable daily intake (ADI) of 10 mg/kg-d established for a combined exposure to MeP and EtP [33], respectively. For PrP, ADI was also less than the reference dose, which is based on antiandrogenicity [34].

## 4. Discussion

**Figure 1:**
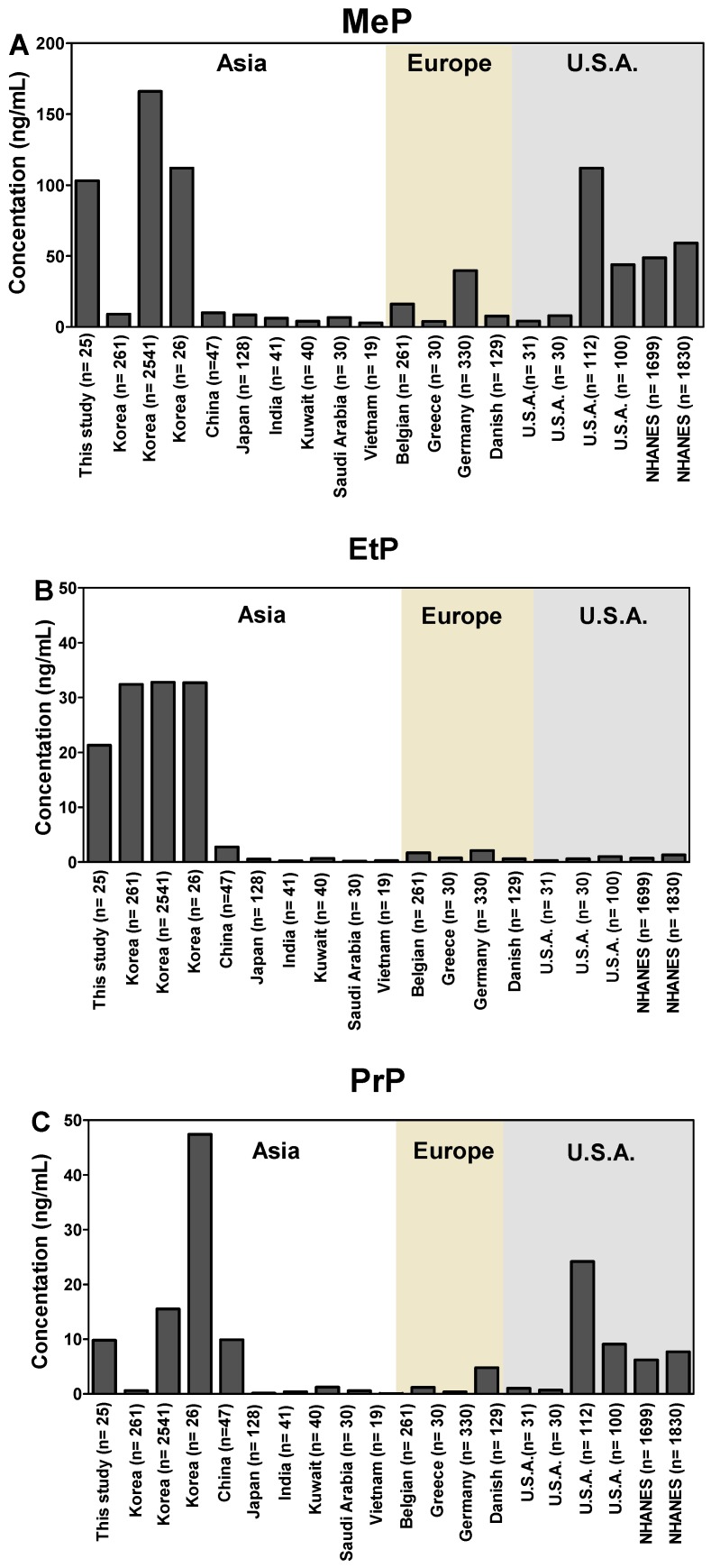
Urinary concentrations of (**A**) methyl paraben (MeP), (**B**) ethyl paraben (EtP), and (**C**) propyl paraben (PrP) in Korea, compared with those reported from other countries. NHANES stands for National Health and Nutrition Examination Survey of U.S.A.

Similar to other reports on populations of Korea, among the temple stay participants, urinary EtP concentrations detected were higher, compared with those of PrP [15,23,25]. The levels of EtP in the urine measured before the temple stay were in a similar range to that reported for other Korean populations, for example, at approximately 30 μg/L. Available information shows that Koreans are exposed to more EtP than other nationalities (Figure 1 and Table S8).

Sharp increases in urinary EtP levels in both genders (Table 1) after the temple stay, that is, >8 times (14.70 vs. 121.78 μg/L) in male and >6 times (12.93 vs. 81.34 μg/L) in female participants, warrant explanation. Though a definitive answer to this observation is not available yet, it is clear that certain factors that are associated with the temple stay program are responsible for the increase of EtP exposure. One such factor could be the diet with which the participants were provided during the temple stay program. Indeed, many traditional condiments or seasoning that are frequently used in vegetarian cooking in Korea, for example, soy sauce, pepper paste (Gochujang), bean paste (Doenjang), and vinegar, could contain parabens [36,37]. While BuP was banned in 2009, MeP, EtP, and BuP are permitted in these sauces and condiments as preservatives in Korea. For example, EtP can be used as a preservative in soy sauce and vinegar at up to 0.25 g/kg and 1 g/L in Korea, respectively (Table S9). Gochujang and soy sauce sold in Korea are reported to contain paraben levels at up to 29.7 mg/kg as EtP and BuP (Table S10) [36]. Compared with other food, the amount of seasonings and condiments consumed is small; the average daily consumption amount of seasonings among general Korean is 34.42 g/day [38]. These sources may contribute significantly to the total body burden of EtP among Korean population. For example, if one should consume soy sauce that contains EtP of 29.7 mg/kg [36], at an average consumption rate of 6.58 g/day [38], assuming average daily urinary excretion of 2 L [16,39] and a urinary excretion fraction (Fue) of 13.7% [16], the EtP concentration in the urine that is contributed by soy sauce consumption is estimated at 13.23 μg/L. While this estimate is about 10 times lower than the average level of EtP measured after the temple stay program (154 μg/L), this is a contribution from the consumption of soy sauce only, and there might be other sources of exposure to EtP in the diets that the temple stay participants consumed. Moreover, Kimchi, a traditional Korean fermented cabbage, was reported to contain 1.9 mg/kg parabens as a sum of EtP and BuP [36,37]. The reason that Kimchi was detected for parabens could be not only owing to seasoning added to Kimchi, but also to fermented cabbage itself. Indeed, available reports suggest production of EtP by natural fermentation of some plants [40,41].

Significant gender differences were observed on the concentrations of MeP, PrP, and BuP in the urine samples (Table 1). In particular, MeP and PrP levels were higher among females, which is consistent with previous reports elsewhere [15,17,26,27,42,43]. In Wang et al. [44], the concentration of total urinary parabens in female was about 10 times higher than male in China. The difference in the urinary concentrations by gender may be the result of the widespread use of MeP and PrP in personal care [45]. Women tend to more frequently use PCPs such as facial cream, body lotion, hand cream, and sunscreen than men [46,47]. In the USA, sunscreen and skin lotion contained the highest levels of parabens among several consumer products, and the measured parabens were mostly MeP and PrP (maximum, MeP: 912 μg/g) [2]. In China, similarly, MeP and PrP occupied >95% of the total parabens measured in PCPs [3]. PCPs use is not an important determinant of EtP exposure, because, although the EtP has been found to be used in some PCPs, its concentration, generally about 100 μg/g, is negligible compared with those of MeP or PrP that were reported in PCPs [3].

It is interesting to note that urinary MeP and PrP levels did not decrease following the temple stay (Table 1). These levels increased slightly, though statistical significance was not observed. Because MeP and PrP are most widely used in personal care products, separately or often in combination [48], the observation of negligible changes in the urinary concentration after the intervention suggests that the use of personal care products, in general, did not change during the temple stay. The negligible changes of urinary benzophenones after the temple stay also support this speculation (Tables S6 and S7). BP-3 is most frequently used in personal care products including sunscreens [31], and BP-1 is a major metabolite of BP-3. We thus assumed that their contribution from the foods would be relatively small. Previously, BP-3 was detected in nearly all tested body lotion (8 in 8 products) and hand lotion (17 in 18 products), not only in sunscreens (12 in 12 products) in China [45,49]. These observations on MeP, PrP, and BPs are different from our previous observation, which reported decreased urinary monoethyl phthalate (MEP) levels in the same population after the temple stay.

**Figure 2:**
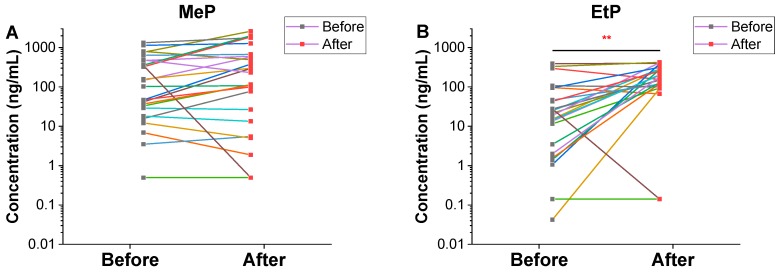

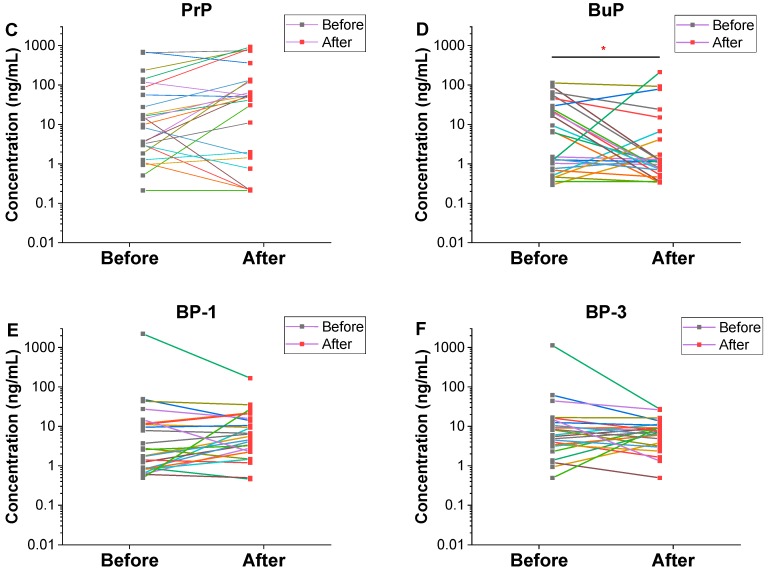
Comparison of urinary paraben and benzophenone concentrations measured before and after the temple stay program for each participant. **A**: methylparaben, **B**: ethylparaben, **C**: propylparaben, **D**: butylparaben (BuP), **E**: benzophenone-1 (BP-1), **F**: benzophenone-3 (BP-3). * Significant differences between before and after the temple stay program (*p* < 0.05). ** indicates statistical difference at *p* < 0.01.

Different patterns of change in urinary chemical concentrations along with an often wide range of variations observed after the temple stay suggest that there might be some exposure sources of these chemicals that were not controlled by the intervention (Figure 2). The use of personal care products is one of such exposure sources that could not be controlled. Because we did not ask the participants for the use pattern of personal care products before and during the temple stay, it is not possible to confirm that contribution of personal care product use on the changes of urinary paraben levels. Further refined study involving the measurements of major exposure sources including personal care products and condiments used in diet, with more rigorous intervention design, is warranted. However, our previous observation on the same population strongly suggests the decrease of the personal care product use during the temple stay. Previously, in the present population, significant decreases of urinary phthalate metabolite levels, including MEP by 10 times among females and 2 times among males, were observed following the temple stay [29]. As a metabolite of diethyl phthalate (DEP), urinary MEP concentrations often indicate the use of personal care products including cosmetics. If the use of PCPs was indeed decreased during the temple stay, a slightly increasing, even though insignificant, pattern of urinary MeP and PrP levels after the temple stay should be explained by other exposure sources, for example, vegetarian diet. Parabens are present naturally in plants as natural antimicrobials, and also produced by natural fermentation of plants [20,40,41]. Contributions of plant-based diets to the urinary levels of MeP and PrP deserve further investigation, employing more rigorous intervention on the use of personal care products and diets.

A significant decrease of urinary BuP concentrations following the temple stay program in male adults (Table 1) suggests that the use of certain male personal care products was reduced through the temple stay. Major sources of BuP exposure among general populations include liquid soap and body wash products among adults [50], and baby wipes, sunscreen, and body lotion among young children [51].

As an intervention study, the present study has several limitations, including a small sample size leading to low statistical power, no information on personal care product use during the temple stay, and a lack of paraben measurement in the actual dietary items during the intervention. However, our unique intervention study design clearly showed that short-term changes in dietary behavior could cause a significant impact on paraben exposure levels, especially on EtP, among the general population of Korea. Studies to investigate dietary contribution and exposure sources of EtP among the Koreans are warranted.

## 5. Conclusions

We investigated the effects of dietary intervention on paraben exposure in a group of the temple stay participants, who followed a strict Buddhist vegetarian diet for five days. Following the temple stay program, urinary levels of EtP increased significantly among the male participants, and those of BuP decreased. Although statistical significance was not observed, among female participants, urinary EtP levels also increased by >6 times following the temple stay. The increase of EtP after the temple stay could be attributable to the vegetarian diets, which include typical Korean seasoning and condiments. Further studies involving actual measurements of diets and more strict intervention design are warranted to confirm the quantification of dietary contribution to the EtP exposure among the Korean population.

## Figures and Tables

**Table 1:** Concentrations (μg/L) and detection frequency of methyl-, ethyl-, propyl-, and butylparaben; benzophenone-1; and benzophenone-3 in urine samples of the participants (*n* = 25) before and after the temple stay.

Chemical	Value	Total		Male (*n* = 16)		Female (*n* = 9)	
		Before	After	Before	After	Before	After
MeP	DF	96.0	92.0	100	93.8	88.9	88.9
	GM	84.9	109	51.2	53.7	208	384
	P50	103	231	44.2	104	481	1270
	P95	1072	1962	551	580	1248	2350
EtP	DF	96.0	92.0	100	93.8	88.9	88.9
	GM	14.0	105	14.7	122	12.9	81.3
	P50	21.3	154	20.1	176 **	42.9	130 *
	P95	323	410	321	400	240	379
PrP	DF	96.0	88.0	100	87.5	88.9	88.9
	GM	11.0	21.1	4.3	7.5	58.9	133.2
	P50	9.80	51.2	3.30	20.9	120	359
	P95	573	838	27.1	127	678	893
BuP	DF	96.0	92.0	100	93.8	88.9	88.9
	GM	4.71	2.06	3.60	1.03	7.57	7.11
	P50	6.40	1.10	3.90	1.10 *	20.4	15.0
	P95	86.3	89.8	64.7	4.80	93.9	164
BP-1	DF	96.0	96.0	100	93.8	88.9	100
	GM	4.12	5.74	2.60	3.78	9.34	12.1
	P50	2.58	5.64	1.58	3.81	9.52	10.5
	P95	47.7	33.7	32.8	17.4	1349	114
BP-3	DF	96.0	96.0	100	93.8	88.9	100
	GM	7.51	6.47	5.38	6.19	13.57	6.99
	P50	6.00	7.30	5.14	7.23	12.5	7.65
	P95	58.5	24.1	48.8	16.7	685	22.8
MeP	DF	96.0	92.0	100	93.8	88.9	88.9
	GM	52.7	54.5	30.4	25.9	140	205
	P50	67.8	85.6	23.9	75.7	243	560
	P95	604	858	347	248	964	1218
EtP	DF	96.0	92.0	100	93.8	88.9	88.9
	GM	8.72	52.6	8.73	58.6	8.70	43.5
	P50	12.1	85.1	11.2	90.6 *	17.4	85.1
	P95	180	204	173.4	171	149	314
PrP	DF	96.0	88.0	100	87.5	88.9	88.9
	GM	6.83	10.5	2.54	3.59	39.7	72.3
	P50	7.80	21.1	1.80	9.40	67.1	131
	P95	321	350	12.0	61.0	507	551
BuP	DF	96.0	92.0	100	93.8	88.9	88.9
	GM	2.93	1.03	2.14	0.49	5.10	3.80
	P50	4.20	0.50	2.40	0.40 *	10.3	5.10
	P95	55.7	26.5	25.5	2.70	61.5	104
BP-1	DF	96.0	96.0	100	93.8	88.9	100
	GM	2.56	2.87	1.54	1.82	6.29	6.44
	P50	1.50	2.37	0.89	1.46	5.08	5.51
	P95	21.5	14.3	10.4	9.07	653	78.6
BP-3	DF	96.0	96.0	100	93.8	88.9	100
	GM	4.67	3.23	3.20	2.98	9.14	3.74
	P50	5.20	2.70	2.64	3.18	7.58	2.63
	P95	16.2	8.46	14.3	7.01	333	14.0

Abbreviations: DF, detection frequency (%); GM, geometric mean; MeP, methyl paraben; EtP, ethyl paraben; PrP, propyl paraben; BuP, butyl paraben; BP-1, benzophenone-1; BP-3, benzophenone-3. * *p* < 0.05 and ** *p* < 0.01 indicate significant differences between intervention phases as determined by non-parametric method (paired-Wilcoxon signed-rank test).

## Supplementary Materials

The following are available online at https://www.mdpi.com/2305-6304/8/1/3/s1, Figure S1. Distribution of urinary parabens among the temple stay participants (unadjusted, *n* = 25). Table S1: Characteristics of study population. Table S2: Tap water (mL/day) and food (servings per day) intake information of study participants before the temple stay (*n* = 25). Table S3: HPLC condition for analysis of urinary chemicals. Table S4: Mass spectrometer conditions for analysis of urinary chemicals. Table S5: Recoveries and precisions of benzophenones and parabens in urine samples. Table S6: Comparison of urinary levels of test chemicals by gender and following the temple stay, based on the mixed effects model. Table S7: Mixed-effects model results from multilevel spline model. Table S8: Concentrations (μg/L) of parabens in the human urines reported worldwide. Table S9: Maximum levels of parabens allowed in food, cosmetics, and personal care products in several countries worldwide. Table S10: Concentrations of parabens reported in condiment and other foodstuff in several Asian countries. Table S11: Estimated daily intake amount of each paraben among the study participants.
