# Supplementary material for: Influence of Vegetarian Dietary Intervention on Urinary Paraben Concentrations: A Pilot Study with ‘Temple Stay’ Participants
Source: Toxics. 2020 Jan 17;8(1):3. doi: 10.3390/toxics8010003 (PMC7151732; doi:10.3390/toxics8010003)
Supplement: Supplementary file 1 [file toxics-08-00003-s001.pdf]

Supplementary Materials: Influence of Vegetarian Dietary Intervention on Urinary Paraben Concentrations: A Pilot Study with 'Temple Stay' Participants

Areum Jo, Sunmi Kim, Kyunghee Ji, Younglim Kho and Kyungho Choi

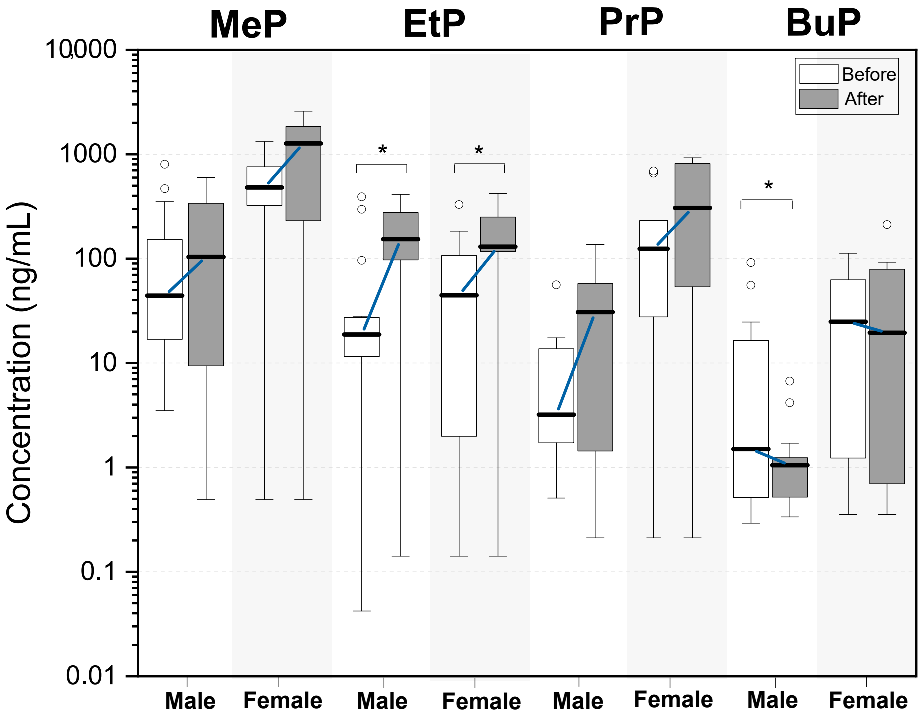

Figure S1. Distribution of urinary parabens among the temple stay participants (unadjusted, n = 25). Bottom and top of a box indicates the first and third quartiles of the distribution, respectively. The black bands inside the boxes show the median. The whiskers represent the 5th and 95th percentiles. * difference between intervention as determined by p-value (<0.05) for non-parametric method (paired-Wilcoxon signed-rank test).

Table S1. Characteristics of study population.

**Characteristic**	***n* (%)**
Age (years)	
10 to 19	3 (12)
20 to 39	11 (44)
40 to <60	9 (36)
≥60	2 (8)
Gender	
Male	16 (64)
Female	9 (36)
Body mass index (kg/m^2)	
<18.5	0 (0)
18.5 to 22.9	16 (64)
>23	9 (36)
Urinary creatinine (mg/dL)[a]	
Pre-intervention	
<118.6	5 (20)
>118.6	20 (80)
Post-intervention	
<118.6	2 (8)
>118.6	23 (92)

[a] Creatinine data are classified by the median (118.6 mg/dL) reported in [1] for a 1988–1994 sample of 22,245 individuals (6–90 years of age).

Table S2. Tap water (mL/day) and food (servings per day) intake information of study participants before the temple stay (*n* = 25).

**Type**	**Gender**	**Median**	**95th**
Tap water	Male	1000.0	3600
	Female	1360.0	1780
Beef	Male	0.30	2.00
	Female	0.15	1.50
Pork	Male	0.40	2.50
	Female	0.45	5.00
Chicken	Male	0.62	1.00
	Female	0.85	2.00
Dairy products	Male	2.25	5.00
	Female	2.00	5.50
Other meats	Male	0.00	0.50
	Female	0.00	0.50
Raw fish (sashimi)	Male	0.00	1.00
	Female	0.00	1.00

None of the subjects indicated the consumption of mutton, goat meat, duck, or turkey during the surveyed period. Source : [2].

Table S3. HPLC condition for analysis of urinary chemicals.

Parameter	Condition
Column	Synergi 4 μ Fusian-RP 75 × 2 mm 4 μm
Mobile phase	A: 0.1% acetic acid in water<br>B: 0.1% acetic acid in ACN
flow rate	400 μL/min (gradient)
Injection volume	5 μL
Ionization mode	ESI negative/positive[a]
Curtain gas	10 psi
Column temperature	40 °C
Ion spray voltage	−4500 V/5500 V[a]
Ion source gas 1	40 psi/30 psi[a]
Ion source gas 2	60 psi/10 psi[a]
Collision gas	5

[a]HPLC condition of benzophenone-3.

Quality control and quality assurance

The accuracy of analysis was tested at three different concentrations of spiked samples, i.e., low (2 μg/L), medium (10 μg/L), and high (50 μg/L). For each concentration of the spiked samples, five replicates were prepared and were measured. Percent recovery was then calculated as a measure of accuracy. Precision of the analytical procedure was measured as the coefficient of variation (CV) divided by the mean of the measured concentrations at a given concentration (Table S4).

Table S4. Mass spectrometer conditions for analysis of urinary chemicals.

Compound	Q1 Mass	Q3 Mass	DP	EP	CE	CXP
$^{13}C_6$-Methylparaben	157	98	−75	−10	−30	−1
$^{13}C_6$-Ethylparaben	171	98	−65	−10	−26	−5
$^{13}C_6$-Propylparaben	185	98	−60	−10	−32	−17
$^{13}C_6$-Butylparaben	199	98	−80	−10	−28	−19
$^{13}C_6$-Benzophenone-1	219	135	−95	−10	−26	−7
Benzophenone-d_{10}	193	110	76	10	25	10
Methylparaben	151	92	−50	−10	−26	−17
Ethylparaben	165	92	−55	−10	−30	−17
Propylparaben	179	92	−60	−10	−30	−17
Butylparaben	193	92	−65	−10	−30	−17
Benzophenone-1	213	135	−100	−10	−22	−23
Benzophenone-3	229	105	61	10	29	10

Abbreviations: DP, Declustering Potential, V; CE, Collision Energy, V; CXP, Collision Cell Exit Potential, V; Q1, Precursor ion mass, m/z; Q3, Daughter ion mass, m/z. EP, Entrance Potential.

Table S5. Recoveries and precisions of benzophenones and parabens in urine samples.

Compound	Spiking Concentration (μg/L)	Recovery Intra-day (*n* = 5)	Recovery Inter-day (*n* = 5)	Precision (CV[a], %) Intra-day (*n* = 5)	Precision (CV[a], %) Inter-day (*n* = 5)
Methylparaben	2	108.0	100.8	8.6	7.4
	10	113.6	100.2	12.4	8.6
	50	109.4	104.3	3.4	3.8
Ethylparaben	2	107.7	97.7	5.6	4.7
	10	97.9	95.7	3.8	4.1
	50	99.5	98.2	1.8	2.5
Propylparaben	2	85.5	84.5	16.7	14.5
	10	91.8	92.7	7.2	5.2
	50	100.1	98.5	5.1	4.2
Butylparaben	2	97.6	101.5	18.8	11.7
	10	91.7	93.1	6.9	6.4
	50	95.4	94.7	2.1	2.1
Benzophenone-1	2	97.6	85.6	8.7	4.9
	10	106.1	109.6	6.4	3.4
	50	99.8	101.0	4.9	6.0
Benzophenone-3	2	108.8	99.4	6.2	7.1
	10	101.9	98.3	2.6	3.0
	50	99.8	98.8	4.8	5.4

[a]Coefficient of variation.

Table S6. Comparison of urinary levels of test chemicals by gender and following the temple stay, based on the mixed effects model.

	Male vs. Female			Pre vs. Post Temple Stay		
**Analyte**	**Slope[a]**	**95% CI for Slope Estimate**	***p***	**Slope[b]**	**95% CI for Slope Estimate**	***p***
MeP	1.93	0.07 to 3.80	**0.04***	0.09	−0.66 to 0.85	0.80
EtP	−0.01	−1.70 to 1.68	0.99	1.91	0.76 to 3.07	**0.00****
PrP	2.85	1.10 to 4.60	**0.00****	0.44	−0.48 to 1.37	0.33
BuP	1.68	0.34 to 3.01	**0.02***	−1.08	−2.06 to −0.10	**0.03***
BP-1	0.85	−0.30 to 2.01	0.14	0.06	−0.43 to 0.54	0.81
BP-3	0.36	−0.54 to 1.26	0.42	−0.36	−0.90 to 0.17	0.18

Abbreviation: CI, Confidence interval; Pre, Pre-intervention, Post, Post-intervention. [a]Slope between gender. [b]Slope between the temple stay. Adjusted for age and log creatinine concentration in the urine. *$p < 0.05$, **$p < 0.01$.

Table S7. Mixed-effects model results from multilevel spline model.

Analyte	Gender	Percent Change (%)[a]	95% CI for Slope Estimate	*p-value*
MeP	Male	−28.02 (51.21 vs. 53.74)	−1.70 to 1.04	0.627
	Female	36.34 (208.33 vs. 384.3)	−2.45 to 3.08	0.811
EtP	Male	561.28 (14.70 vs. 121.78)	0.35 to 3.43	**0.018***
	Female	425.93 (12.93 vs. 81.34)	−1.05 to 4.37	0.210
PrP	Male	12.42 (4.28 vs. 7.46)	−1.23 to 1.47	0.860
	Female	70.65 (58.89 vs. 133.15)	−2.23 to 3.30	0.685
BuP	Male	−75.66 (3.60 vs. 1.03)	−2.52 to −0.31	**0.014***
	Female	−34.16 (7.57 vs. 7.11)	−2.83 to 1.99	0.715
BP-1	Male	7.38 (2.60 vs. 3.78)	−0.53 to 0.67	0.811
	Female	2.33 (9.34 vs. 12.06)	−2.09 to 2.14	0.981
BP-3	Male	−16.36 (5.38 vs. 6.19)	−0.87 to 0.52	0.603
	Female	−52.29 (13.57 vs. 6.99)	−2.29 to 0.80	0.318

Abbreviation: CI, Confidence interval; Pre, Pre-intervention, Post, Post-intervention. [a]Percent change in the GM between the two time periods, with GMs of the two time periods shown in parentheses. Adjusted for age and log creatinine concentrations. *$p < 0.05$.

Table S8. Concentrations (µg/L) of parabens in the human urines reported worldwide.

Country	Sampling Year	*N*	Population	Age (Years)	Median (µg/L) (95th Percentile)				Reference
					MeP	EtP	PrP	BuP	
Korea	2007	16	Males	14–64	44.2 (551)	20.1 (320.5)	3.3 (27.1)	3.9 (64.7)	This study
		16		14–64	104 (579.5)	175.5 (399.8)	20.9 (127)	1.1 (4.8)	
		9	Females	13–47	481 (1248)	42.9 (240.2)	120 (677.8)	20.4 (93.92)	
		9		13–47	1270 (2350)	130 (378.8)	359 (893.2)	15 (164.2)	
		25	All	13–64	103 (1072)	21.3 (154)	9.8 (572.8)	6.4 (86.34)	
		25		13–64	231 (1962)	154 (410.2)	51.2 (837.6)	1.1 (89.84)	
	2015	108	Males	27.5–36.4	9.10 (31.6)[a]	32.6 (80)[a]	<LOD (2.7)[a]	<LOD (<LOD)[a]	[3]
		108		27.5–36.4	11.1 (48.2)[a]	19.4 (67.1)[a]	0.8 (5.9)[a]	<LOD (<LOD)[a]	
		153	Females	27.5–36.4	11.6 (52.1)[a]	31.4 (90.1)[a]	0.9 (10.7)[a]	<LOD (1.4)[a]	
		153		27.5–36.4	19.2 (69.5)[a]	19.2 (56.8)[a]	1.8 (11)[a]	<LOD (<LOD)[a]	
		261	All	27.5–36.4	9.9 (39.8)[a]	32.4 (82.8)[a]	0.6 (6.2)[a]	<LOD (<LOD)[a]	
		261		27.5–36.4	15.8 (62)[a]	19.2 (62.5)[a]	1.5 (8.8)[a]	<LOD (<LOD)[a]	
	2009–2010	1167	Males	3–69	123 (-)	37.9 (-)	5 (-)	0.33 (-)	[4]
		1374	Females	3–69	233 (-)	29.2 (-)	37.8 (-)	1.48 (-)	
		2541	All	3–69	166 (1145)	32.8 (467)	15.5 (300)	0.51 (57.4)	
	2006–2007	26	All	30	112 (1440)	32.7 (113)	47.4 (421)	4.30 (46.7)	[5]
China	2010–2012	47	All	2–67	10.1 (883)	2.74 (67.1)	9.91 (357)	<LOQ (1.59)	
India	2010–2012	41	All	24–75	6.28 (96.4)	0.25 (11.1)	0.39 (44.4)	<LOQ (0.9)	
Japan	2012–2013	128	Females	19–22	273 (4107)[b]	4.04 (166)[b]	8.39 (441)[b]	0.634 (73.7)[b]	[6]
	2010–2012	36	All	2–59	8.65 (298)	0.52 (98.5)	0.13 (98.3)	<LOQ (18.7)	[5]
Kuwait	2010–2012	40	All	6–73	4.03 (137)	0.68 (47.6)	1.24 (50)	<LOQ (37.1)	
Saudi Arabia	2012	30	All	3–87	6.80 (99,3)	0.19 (4.5)	0.58 (30.4)	<LOQ (0.78)	
Vietnam	2010–2012	19	All	19–83	2.92 (72.8)	0.26 (1.31)	0.07 (35.6)	-	
Belgium	2013	123	Males	1–85	7.70 (223.4)	1.30 (41.4)	0.50 (20.2)	<LOD (4.9)	[7]
		138	Females	1–85	32.4 (630.6)	1.9 (83.1)	3.30 (116.5)	0.50 (11.1)	
		261	All	1–85	16.1 (462.6)	1.7 (67.7)	1.2 (78.8)	<LOD (8)	
Denmark	2006	60	Males	19.7	17.7 (64.6)[a]	1.98 (5.35)[a]	3.60 (14)[a]	0.19 (1.01)[a]	[8]

Country	Sampling Year	*N*	Population	Age (Years)	Median (μg/L) (95th Percentile)				Reference
					MeP	EtP	PrP	BuP	
		65	Males	5–20	4.65 (64.4)	0.58 (13.8)	0.67 (20.2)	-(2.4)	
	2007	64	Females	5–20	10.4 (555)	0.52 (40.2)	1.85 (84.8)	-(20.6)	[9]
		129	All	5–20	7.7 (251)	0.58 (13.8)	1.02 (46.2)	-(5.24)	
Denmark	2007–2009	130	Males	18.9	12.7 (383)	1.35 (37.6)	2.72 (127)	0.22 (27.1)	[10]
Germany	1995–2012	660	Males	20–30	23.2 (224)	1.2 (17.7)	1.5 (46)	<LOQ (5.4)	[11]
	1995–2012	330	Females	20–30	57.2 (388)	4.1 (51.5)	9.5 (122)	0.9 (12.7)	[11]
		330	All	20–30	39.8 (319)	2.1 (39.1)	4.8 (74)	<LOQ (10.7)	
	2010–2011	39	Males	18–64	20.7 (231)	1.3 (47.1)	0.8 (25.8)	<LOQ (17.7)	[12]
	2007–2009	59	Females	29–48	51.4 (609)	4.2 (44)	3.9 (79.4)	0.6 (16.9)	[12]
Greece	2012	30	All	23–75	4.01 (208)	0.76 (24.5)	0.37 (112)	<LOQ (5.54)	[5]
Tunisia	2012	34	Females	≥18	34.94 (80.91)[a]	1.77 (9.90)[a]	3.06 (12.54)[a]	<LOD (5.15)[a]	[13]
U.S.A.	2010–2012	31	All	11–66	4.23 (123)	0.30 (12.6)	0.69 (24.8)	<LOQ (3.88)	[5]
	2004–2015	383	Males	18–55	23.2 (80.4)[a]	-	2.30 (12.1)[a]	<LOD (0.30)[a]	[14]
	2011	30	All	-	8 (240)[b]	0.58 (23.5)[b]	-	-	[15]
	2004–2010	245	Male	23.9–56.8	29 (96.7)[a]	-	3.10 (16.80)[a]	<LOD (0.50)[a]	
		408	Females	20.9–46.7	155 (422)[a]	-	34.3 (118)[a]	1.20 (7.65)[a]	[16]
		653	All	20.9–56.8	112 (354)[a]	-	24.2 (90.2)[a]	0.70 (5.40)[a]	
	2005–2007	143	Females	18–44	59 (552.7)	1.1 (49.4)	15.1 (202.4)	0.5 (17.9)	[17]
	2003–2005	100	All	-	43.9 (680)	1.0 (47.5)	9.1 (279)	0.5 (29.5)	[18]
NHANES[c]	2011–2012	1,699	All	>30	48.80 (188)[a]	0.71 (7.10)[a]	6.2 (35.5)[a]	0.14 (0.4)[a]	[19]
	2009–2010	1,830	All	>30	59.20 (241)[a]	1.30 (8.7)[a]	7.70 (49.9)[a]	0.14 (0.8)[a]	[19]
	2009–2010	415	Females	18–40	119 (1,269)	-	24.8 (434)	0.4 (22.1)	[20]
	2007–2008	365	Females	18–40	146 (1,444)	-	33.6 (410)	0.7 (33.6)	[20]

Abbreviations: LOD, Limit of detection; LOQ, Limit of quantification; -, not available. [a] 75th percentiles. [b] max. [c] NHANES, National Health and Nutrition Survey conducted by the U.S. Centers for Disease Control and Prevention (CDC).

Table S9. Maximum levels of parabens allowed in food, cosmetics and personal care products in several countries worldwidde.

Country	Category	Chemicals	Type	Criteria	Reference
Korea	Food	MeP and EtP	Capsule and jam	1.0 g/kg	[21]
			Mango chutney and soy sauce	0.25 g/kg	
			Vinegar and beverage	1.0 g/L	
			Sauces	0.2 g/kg	
			Fruit and vegetable peel	0.012 g/kg	
	Cosmetic product	Single ester and mixture		0.4%, 0.8%	
	Quasi-drugs	MeP and PrP	Toothpaste	<0.2%	
			Oral pharmaceutical liquid	<0.01%	
			Oral cleansing wipes	<0.01%	
Japan	Cosmetic product	*p*-hydroxybenzoic acid ester		1.00%	[22]
EU	Food	Single ester and mixture (MeP, EtP, PrP, BuP)	Snacks - Potato, cereal, flour or starch-based and processed nuts (including coated nuts and nut mixtures)	0.3 g/kg	[23]
			Confectionery (excluding chocolate)	0.3 g/kg	
			Liquid dietary food supplements	2 g/kg	
			Jelly coatings of meat products (cooked, cured or dried)	1 g/kg	
		MeP and EtP		0.3–2 g/kg	
		Other parabens		Prohibited	
	Cosmetic product	Single ester and mixture		0.4%, 0.8%	[24]
	Baby product	PrP and BuP	Products of diaper wearing area of infant (<3 years old)	Prohibited	[25]
Danish	Personal care product	PrP, BuP and their isoforms and salts	Products (<3 years old)	Prohibited	[26]
U.S.	Food	MeP and PrP		< 0.1%	[27]

Table S10. Concentrations of parabens reported in condiment and other foodstuff in several Asian countries.

Country	Food Groups	Analyte	Food Type	N	DR (%)	Concentration (mg/kg)	Reference
Korea	Beverage	MeP, EtP, PrP, BuP	Functional beverage, green tea, black tea, red ginseng	61	9.8	2.7	[28,29]
	Condiments	EtP, BuP	Soy sauce	46	43.5	29.7	
		BuP	Gochujang	12	8.3	2.9	
		BuP, isoBuP, isoPrP	Sauces	19	31.6	22.4	
	Kimchi, pickles	BuP	Pickle	80	6.3	1.9	
	Condiments	BuP, iso-BuP, EtP, PrP, iso-PrP	Soy sauce	75	68	54.6	[30]
			Seasoning	18	0	-	
			Vinegar	69	0	-	
	Beverage		Mixed beverage	42	0	-	
	Beverage		Ginseng	6	0	-	
	Beverage		Red ginseng	9	0	-	
	Beverage		Fruit and vegetable juice (except unheated juice)	75	0	-	
	Liquors			12	25	20.7	
	Vegetable		Surface of vegetable	42	0	-	
Japan	Beverage	EtP, PrP, iso-PrP, BuP, n-BuP	Nonalcoholic beverages	1592	14	27	[31]
	Condiments		Soy sauce	1260	49.8	69	
	Condiments		Fruit sauce	148	16.9	86	
	Condiments		Vinegar	31	9.7	45	
	Condiments		Syrup	24	37.5	63	
	Fruits and vegetables			46	0	0	
	Non-permissible foods*		Non-permissible foods	6775	0.2	39	
	Breakdown of non-permissible foods		Preserved foods boiled down in soy sauce, "Tsukudani"	270	0.7	25	
	Condiments		Condensed seasoned soy sauce, "Tare"	369	0.8	23	
	Condiments		Soy sauce-pickled foods, "Shoyu-zuke"	42	11.9	56	
	Condiments	EtP, PrP, iso-PrP, BuP, n-BuP	Sauce other than fruit sauce	20	5	20	
	Condiments		Processed soy sauce, "Shoyu-kakouhin"	3	100	30	
	Beverage		Latic acid bacteria beverages	48	2.1	20	
Taiwan	Condiments	EtP	A grate soy sauce	10	10	86.4[a]	[32]

Country	Food Groups	Analyte	Food Type	N	DR (%)	Concentration (mg/kg)	Reference
Taiwan	Condiments	PrP	A grate soy sauce	10	10	54.2 [a]	[32]
	Condiments	BuP	A grate soy sauce	10	60	33.4–142.4 [a]	
	Condiments	EtP	Fish sauce	4	25	97.1 [a]	
	Condiments	BuP	Fish sauce	4	25	32.4 [a]	
China	Condiments	EtP	High salt constant temperature fermentation	-	-	12.1	[33]
	Condiments	MeP	Soy sauce (10), vinegar (9), cooking wine (3), ketchup (2), bean paste (7), starch (3), aniseed (3), chili powder (3), dried soup materials (4), honey (1), oyster sauce (1), sugar (1)	47	100	0.02	[34]
		EtP			87	0.043	
		PrP			79	0.012	
		BuP			57	0.0002	

* non-permissible foods: foods in which preservatives are not permitted to be used in Japan [31].

Table S11. Estimated daily intake amount of each paraben among the study participants.

Chemical	Gender	UC (μg/L)	F_{ue}	EDI (μg/kg bw-d)					
				Median	SD	Min	25th	75th	Max
MeP	Male	104	0.174	17.46	35.89	0.08	2.40	55.29	109.29
	Female	1270		224.58	187.21	0.10	53.10	386.62	476.53
EtP	Male	175.5	0.137	37.17	27.84	0.03	25.12	58.99	95.93
	Female	130		39.54	24.96	0.04	30.41	66.62	78.98
PrP	Male	20.9	0.102	6.09	11.96	0.05	0.45	16.50	38.10
	Female	359		108.30	145.74	0.07	21.10	300.89	378.27
BuP	Male	1.1	0.056	0.52	0.88	0.13	0.39	0.74	3.15
	Female	15		9.74	50.89	0.23	0.47	42.35	157.74

Estimated daily intake (EDI) of each paraben was calculated following Moos, *et al.* [20]).: EDI = UC × UV_{24h}/F_{ue} × bw. UC: urinary concentration (μg/L). EDI was calculated using urinary concentration of each participant after the temple stay. Fue: urinary excretion fraction. UV_{24h}: the total volume of urine in 24 h (assumed 2 L). BW: body weight of each participant of the present study.

References

1. Barr, D.B.; Wilder, L.C.; Caudill, S.P.; Gonzalez, A.J.; Needham, L.L.; Pirkle, J.L. Urinary creatinine concentrations in the US population: implications for urinary biologic monitoring measurements. *Environmental health perspectives* **2005**, *113*, 192.
2. Ji, K.; Lim Kho, Y.; Park, Y.; Choi, K. Influence of a five-day vegetarian diet on urinary levels of antibiotics and phthalate metabolites: a pilot study with "Temple Stay" participants. *Environ Res* **2010**, *110*, 375-382, doi:10.1016/j.envres.2010.02.008.
3. Kim, S.; Lee, S.; Shin, C.; Lee, J.; Kim, S.; Lee, A.; Park, J.; Kho, Y.; Moos, R.K.; Koch, H.M. Urinary parabens and triclosan concentrations and associated exposure characteristics in a Korean population− A comparison between night-time and first-morning urine. *International Journal of Hygiene and Environmental Health* **2018**.
4. Kang, H.S.; Kyung, M.S.; Ko, A.; Park, J.H.; Hwang, M.S.; Kwon, J.E.; Suh, J.H.; Lee, H.S.; Moon, G.I.; Hong, J.H., et al. Urinary concentrations of parabens and their association with demographic factors: A population-based cross-sectional study. *Environ Res* **2016**, *146*, 245-251, doi:10.1016/j.envres.2015.12.032.
5. Honda, M.; Morgan, R.; Kannan, K. Parabens in human urine from several Asian countries, Greece, and the United States. *Chemosphere* **2018**.
6. Nishihama, Y.; Yoshinaga, J.; Iida, A.; Konishi, S.; Imai, H.; Yoneyama, M.; Nakajima, D.; Shiraishi, H. Association between paraben exposure and menstrual cycle in female university students in Japan. *Reproductive Toxicology* **2016**, *63*, 107-113.
7. Dewalque, L.; Pirard, C.; Charlier, C. Measurement of urinary biomarkers of parabens, benzophenone-3, and phthalates in a Belgian population. *Biomed Res Int* **2014**, *2014*, 649314, doi:10.1155/2014/649314.
8. Frederiksen, H.; Jorgensen, N.; Andersson, A.M. Parabens in urine, serum and seminal plasma from healthy Danish men determined by liquid chromatography-tandem mass spectrometry (LC-MS/MS). *J Expo Sci Environ Epidemiol* **2011**, *21*, 262-271, doi:10.1038/jes.2010.6.
9. Frederiksen, H.; Jensen, T.K.; Jørgensen, N.; Kyhl, H.B.; Husby, S.; Skakkebæk, N.E.; Main, K.M.; Juul, A.; Andersson, A.-M. Human urinary excretion of non-persistent environmental chemicals: an overview of Danish data collected between 2006 and 2012. Reproduction 2014, 147, 555-565.
10. Joensen, U.N.; Jorgensen, N.; Thyssen, J.P.; Petersen, J.H.; Szecsi, P.B.; Stender, S.; Andersson, A.M.; Skakkebaek, N.E.; Frederiksen, H. Exposure to phenols, parabens and UV filters: Associations with loss-of-function mutations in the filaggrin gene in men from the general population. *Environ Int* **2017**, *105*, 105-111, doi:10.1016/j.envint.2017.05.013.
11. Moos, R.K.; Koch, H.M.; Angerer, J.; Apel, P.; Schroter-Kermani, C.; Bruning, T.; Kolossa-Gehring, M. Parabens in 24 h urine samples of the German Environmental Specimen Bank from 1995 to 2012. *Int J Hyg Environ Health* **2015**, *218*, 666-674, doi:10.1016/j.ijheh.2015.07.005.

12. Moos, R.K.; Angerer, J.; Wittsiepe, J.; Wilhelm, M.; Bruning, T.; Koch, H.M. Rapid determination of nine parabens and seven other environmental phenols in urine samples of German children and adults. *Int J Hyg Environ Health* **2014**, *217*, 845-853, doi:10.1016/j.ijheh.2014.06.003.
13. Jimenez-Diaz, I.; Artacho-Cordon, F.; Vela-Soria, F.; Belhassen, H.; Arrebola, J.P.; Fernandez, M.F.; Ghali, R.; Hedhili, A.; Olea, N. Urinary levels of bisphenol A, benzophenones and parabens in Tunisian women: A pilot study. *Sci Total Environ* **2016**, *562*, 81-88, doi:10.1016/j.scitotenv.2016.03.203.
14. Nassan, F.L.; Coull, B.A.; Gaskins, A.J.; Williams, M.A.; Skakkebaek, N.E.; Ford, J.B.; Ye, X.; Calafat, A.M.; Braun, J.M.; Hauser, R. Personal Care Product Use in Men and Urinary Concentrations of Select Phthalate Metabolites and Parabens: Results from the Environment And Reproductive Health (EARTH) Study. *Environ Health Perspect* **2017**, *125*, 087012, doi:10.1289/EHP1374.
15. Wang, L.; Kannan, K. Alkyl protocatechuates as novel urinary biomarkers of exposure to p-hydroxybenzoic acid esters (parabens). *Environ Int* **2013**, *59*, 27-32, doi:10.1016/j.envint.2013.05.001.
16. Smith, K.W.; Braun, J.M.; Williams, P.L.; Ehrlich, S.; Correia, K.F.; Calafat, A.M.; Ye, X.; Ford, J.; Keller, M.; Meeker, J.D., et al. Predictors and variability of urinary paraben concentrations in men and women, including before and during pregnancy. *Environ Health Perspect* **2012**, *120*, 1538-1543, doi:10.1289/ehp.1104614.
17. Pollack, A.Z.; Perkins, N.J.; Sjaarda, L.; Mumford, S.L.; Kannan, K.; Philippat, C.; Wactawski-Wende, J.; Schisterman, E.F. Variability and exposure classification of urinary phenol and paraben metabolite concentrations in reproductive-aged women. *Environ Res* **2016**, *151*, 513-520, doi:10.1016/j.envres.2016.08.016.
18. Ye, X.; Bishop, A.M.; Reidy, J.A.; Needham, L.L.; Calafat, A.M. Parabens as urinary biomarkers of exposure in humans. *Environmental health perspectives* **2006**, *114*, 1843.
19. Ferguson, K.K.; Colacino, J.A.; Lewis, R.C.; Meeker, J.D. Personal care product use among adults in NHANES: associations between urinary phthalate metabolites and phenols and use of mouthwash and sunscreen. *J Expo Sci Environ Epidemiol* **2017**, *27*, 326-332, doi:10.1038/jes.2016.27.
20. Meeker, J.D.; Cantonwine, D.E.; Rivera-González, L.O.; Ferguson, K.K.; Mukherjee, B.; Calafat, A.M.; Ye, X.; Anzalota Del Toro, L.V.; Crespo-Hernández, N.; Jiménez-Vélez, B. Distribution, variability, and predictors of urinary concentrations of phenols and parabens among pregnant women in Puerto Rico. *Environmental science & technology* **2013**, *47*, 3439-3447.
21. Ministry of Food and Drug Safety, 2016. Announcement. Standards and specifications for food additives. Available online: https://www.foodsafetykorea.go.kr/portal/safefoodlife/notification.do?ntfc_dvs=NTFC02&menu_grp=MENU_NEW04&menu_no=2889menu_grp=MENU_NEW04&menu_no=2889/ (assessed 19 May 2018).
22. Pharmaceuticals and Medical Devices Agency (PMDA). Japan. 2000. The Pharmaceutical Affairs Act. Available online: https://www.mhlw.go.jp/file/06-Seisakujouhou-11120000-Iyakushokuhinkyoku/keshouhin-standard.pdf/ (assessed 13 April 2018).
23. Commission regulation (EU) No 1129. 2011 of 11 November 2011 amending Annex II to regulation (EC) No 1333/2008 of the European Parliament and of the Council by establishing a Union list of food additives Available online: https://op.europa.eu/en/publication-detail/-/publication/28cb4a37-b40e-11e3-86f9-01aa75ed71a1/language-en (accessed on 22 September 2018).
24. Buzek, J., and B. Ask. "Regulation (EC) No 1223/2009 of the European Parliament and of the Council of 30 November 2009 on cosmetic products." Official Journal of the European Union L 342 (2009). Available online: http://data.europa.eu/eli/reg/2009/1223/oj (accessed on 13 September 2018).
25. Regulation, C. "Amending Annexes II and V to Regulation (EC) No 1223/2009 of the European Parliament and of the Council on cosmetic products. In 358/2014." Official Journal of the European Union (2014). http://data.europa.eu/eli/reg/2014/1004/oj (accessed on 23 May 2018).
26. Danish Environmental Protection Agency (EPA). Survey of parabens. Miljøstyrelsen Strandgade 29 1401 København K. 2012. Available online: https://www2.mst.dk/Udgiv/publications/2013/04/978-87-93026-02-5.pdf (accessed on 15 January 15, 2020).
27. U.S. Food and Drug Administration, 2017. CFR-code of federal regulations title 21. Current Good Manufacturing Practice for Finished Pharmaceuticals Part 2017; 211. Available online: https://www.accessdata.fda.gov/scripts/cdrh/cfdocs/cfcfr/CFRSearch.cfm?fr=184.1670 (accessed on 30 March 2018).

28. Choi, S.H., Lee, J.Y., Park, E.Y., Won, J., Hong, K. K., Moon, G.I., Kim, M.S., Hong, J.H. Assessment of estimated daily intakes of preservatives in the Korean population. *Korean J. Food Sci. Technol*. **2008**, *40*, 503–509.
29. Ministry of Food and Drug Safety, 2007. Dietary Intake of Food Additive by Korean Population - Preservatives, Antioxidant. Available online: http://www.ndsl.kr/ndsl/search/detail/report/reportSearchResultDetail.do?cn=TRKO201700002026/ (assessed 10 May 2018).
30. Kim, H.M., Lee, Y.J., Hong, K.H., Ha, S.C., Ahn, M.S., Jo, J.S., Kim, K.S. Intake of food additives in foods by total diet. *Korean J. Food Sci. Technol*. **1998**, *30*, 767–774.
31. Ishiwata, H., NishijimaI, M., Fukasawa, Y., Ito, Y., Yamada, T. Evaluation of preservatives contents in foods and the daily intake deduced from the results of the official inspection in Japan in FY 1994. *Food Hygiene and Safety Science (Shokuhin Eiseigaku Zasshi)*. **1997**, *38*, 145–154.
32. Lin, H.J., Choong, Y.M. A simple method for the simultaneous determination of various preservatives in liquid foods. *J. Food Drug Anal*. **1999**, *7*, 291–304.
33. Zheng, J., Wu, C.D., Huang, J., Zhou, R.Q., Liao, X.P. Analysis of volatile compounds in Chinese soy sauces moromi cultured by different fermentation processes. *Food Sci. Biotechnol*. **2013**, *22*, 605–612.
34. Liao, C., Chen, L., Kannan, K. Occurrence of parabens in foodstuffs from China and its implications for human dietary exposure. *Environ. Int.* **2013**, *57*, 68–74.
